# Direct effects of remote ischemic preconditioning on post-exercise-induced changes in kynurenine metabolism

**DOI:** 10.3389/fphys.2024.1462289

**Published:** 2024-11-26

**Authors:** Paulina Brzezińska, Jan Mieszkowski, Błażej Stankiewicz, Tomasz Kowalik, Joanna Reczkowicz, Bartłomiej Niespodziński, Aleksandra Durzyńska, Konrad Kowalski, Andżelika Borkowska, Jędrzej Antosiewicz, Andrzej Kochanowicz

**Affiliations:** ^1^ Department of Gymnastics and Dance, Gdańsk University of Physical Education and Sport, Gdańsk, Poland; ^2^ Faculty of Physical Education and Sport, Charles University, Prague, Czechia; ^3^ Department of Theory and Methodology of Physical Education and Sport, Faculty of Health Sciences and Physical Education, Kazimierz Wielki University, Bydgoszcz, Poland; ^4^ Department of Bioenergetics and Physiology of Exercise, Medical University of Gdańsk, Gdańsk, Poland; ^5^ Department of Biological Foundations of Physical Education, Faculty of Health Sciences and Physical Education, Kazimierz Wielki University, Bydgoszcz, Poland; ^6^ Faculty of Health Sciences, University of Lomza, Łomża, Poland

**Keywords:** kynurenic acid, limb ischemia, long-distance runner, neuro-protective metabolites, tryptophan, xanthurenic acid

## Abstract

**Purpose:**

Tryptophan (TRP) degradation through the kynurenine pathway is responsible for converting 95% of free TRP into kynurenines, which modulate skeletal muscle bioenergetics, immune and central nervous system activity. Therefore, changes in the kynurenines during exercise have been widely studied but not in the context of the effects of remote ischemic preconditioning (RIPC). In this study, we analyzed the effect of 14-day RIPC training on kynurenines and TRP in runners after running intervals of 20 × 400 m.

**Methods:**

In this study, 27 semi-professional long-distance runners were assigned to two groups: a RIPC group performing 14 days of RIPC training (n = 12), and a placebo group, SHAM (n = 15). Blood was collected for analysis before, immediately after, and at 6 h and 24 h after the run.

**Results:**

After the 14-day RIPC/SHAM intervention, *post hoc* analysis showed a significantly lower concentration of XANA and kynurenic acid to kynurenine ratio (KYNA/KYN) in the RIPC group than in the SHAM group immediately after the running test. Conversely, the decrease in serum TRP levels was higher in the RIPC population.

**Conclusion:**

RIPC modulates post-exercise changes in XANA and TRP levels, which can affect brain health, yet further research is needed.

## 1 Introduction

Remote ischemic preconditioning (RIPC), a procedure in which brief cycles of limb ischemia and reperfusion are induced by inflating and deflating a blood pressure cuff, has attracted attention as a possible support for sports training. Improvements in sports performance have been observed after RIPC (Caru et al., 2019). Several clinical studies have demonstrated its beneficial impact. RIPC has been shown to reduce the markers of heart and liver damage after coronary artery bypass (Venugopal et al., 2009) and liver transplants (Kanoria et al., 2017), respectively. However, the protective mechanisms underlying RIPC are not thoroughly understood. For instance, RIPC has been shown to induce erythropoietin, which provides protective benefits to cardiac tissue. However, data pertaining to the effects of RIPC on metabolism are limited. Tryptophan (TRP) is an essential amino acid that cannot be synthesized during human metabolism and must be supplied through diet (Richard et al., 2009). The required daily dose of L-TRP is 3.5 mg per kg of body mass. Human intake of TRP mainly occurs through consuming dairy products, dark chocolate, eggs, and fish (Friedman, 1996). In humans, TRP is poorly stored by tissues, and solely free plasma TRP is transferred by the large amino acid transporter through the blood-brain barrier. TRP transported to the brain is metabolized into neuroactive TRP metabolites (Agudelo et al., 2014).

Primary TRP metabolites include kynurenine [KYN] (Saran et al., 2021) and monoaminergic neurotransmitters (such as serotonin and melatonin). These metabolites are competitively synthesized via two different pathways. Neuro-protective [kynurenic acid (KYNA), picolinic acid (PA) and xanthurenic acid (XANA)] and neuro-toxic [quinolinic acid (QA), 3-hydroxykynurenin] metabolites are produced during the KYN pathway (Savitz, 2020; Tanaka et al., 2021). The synthesis of specific kynurenine pathway metabolites is tightly regulated and may considerably vary under physiological and pathological conditions. Experimental data consistently imply that shift of the kynurenine pathway to the neurotoxic branch producing QA and 3-HK formation, with a relative or absolute deficiency of KYNA, is a one of the important factors contributing to neurodegeneration. In some diseases increase concentration of KYNA in serum and cerebrospinal fluid is observed (Savitz, 2020; Tanaka et al., 2021). Regarding that penetration of blood brain barrier by KYNA is very poor its serum level reflects formation outside central nervous system and cannot be consider neuroprotective (Agudelo et al., 2014).

Monoaminergic neurotransmitters are synthesized through the serotonergic pathway (Tanaka et al., 2021). The liver-specific enzyme TRP 2,3-dioxygenase (TDO) plays a significant role in the metabolism of TRP, converting over 90% of TRP in the KYN pathway (Cervenka et al., 2017). However, in the lungs, kidneys, spleen, placenta, and blood cells, this reaction is catalyzed by indoleamine-2,3-dioxygenases 1 and 2 [IDO1 and IDO2] (Strasser et al., 2017). IDO typically exhibits minimal activity under normal conditions; however, in the presence of inflammatory signals, its activity significantly increases (Chen and Guillemin, 2009; Yilmaz and Gokmen, 2020; Roth et al., 2021). The enzymatic activity of TDO and IDO is low in the skeletal muscle; therefore, during exercise, it is likely that KYN is produced extramuscularly and is subsequently imported into the skeletal muscle. KYN can be metabolized into KYNA, anthranilic acid (AA), QA, 3-hydroxy-L-KYN (3-HK), XANA, and PA within the skeletal muscle and other tissues. One of the characteristics of adaptation to endurance training is an increase in KYN transaminase (KAT) expression, which catalyzes the conversion of KYN to KYNA (Agudelo et al., 2014; Schlittler et al., 2016; Metcalfe et al., 2018). The transformation of KYN to KYNA serves as a safeguard mechanism against the detrimental effects of KYN on the central nervous system (Agudelo et al., 2014). KYN acts as an agonist of cytosolic transcription factors, such as the aryl hydrocarbon receptor [AhR] (Opitz et al., 2011). KYNA is an antagonist of the glutamate receptors and acts predominantly on the N-methyl-D-aspartate (NMDA) receptor (DiNatale et al., 2010). KAT’s role in producing KYNA helps maintain a balance between excitatory neurotransmitters (glutamate) and inhibitory ones (such as GABA) by inhibiting NMDA receptors, thus reducing glutamate activity in synapses, providing neuroprotective effects and preventing excessive neuronal excitation (excitotoxicity), which can lead to cell damage (Phillips et al., 2019).

Furthermore, KATs are involved in PGC-1α1 regulation mechanism of enhancing the functionality of the malate-aspartate shuttle. This results in a significant enhancement in skeletal muscle bioenergetics (Agudelo et al., 2019); however, the production of QA may have negative consequences. QA can potentially trigger the formation of reactive oxygen species and possesses neuro-toxic properties by activating the NMDA receptor. Additionally, it can disrupt the balance of glutamate release and uptake by neurons and astrocytes and induce lipid peroxidation (Guillemin, 2012; Vecsei et al., 2013; Pukoli et al., 2021).

TRP metabolism can be altered by inflammatory cytokines. Excessive activation of IDO can be induced by pro-inflammatory cytokines, e.g., the interferons alpha (IFN-α) and gamma (IFN-γ), interleukin-1 beta (IL-1β), and tumor necrosis factor-alpha [TNF-α] (Tanaka et al., 2020). Long-distance running has been shown to induce inflammation; moreover, this may affect TRP metabolism.

Recently, we demonstrated that ultramarathon-induced KYN changes could be reduced by vitamin D supplementation (Mieszkowski et al., 2022). We concluded that a decrease in inflammation mediated by vitamin D supplementation was partially responsible for the lower increase in KYN levels observed in athletes after an ultramarathon, when compared with placebo-treated athletes (Mieszkowski et al., 2022).

Some TRP metabolites exert deleterious protective effects on several tissues. Additionally, the protective action of skeletal muscle remote ischemic preconditioning has been well documented; however, its effect on TRP metabolism has not yet been studied. Thus, the purpose of this study was to evaluate whether a 2-week remote ischemic preconditioning training regime could modify post-exercise-induced changes in TRP metabolism.

## 2 Materials and methods

### 2.1 Experimental overview

This study was a double-blind, randomized controlled trial with parallel groups. Participants were randomly assigned to two study groups that underwent either remote ischemic preconditioning (RIPC) or SHAM-controlled intervention (placebo intervention) (RIPC vs. SHAM) for 14 consecutive days. During the first early-morning visit (pre-intervention) to the laboratory, venous blood samples were drawn, and basic anthropometric characteristics were measured, such as age, height, and bioimpedance body composition (InBody 720, South Korea, Seoul). The following day, a 20 × 400 m running trial (running test - RT) was performed with the intensity increasing during each stretch of the run (according to the running trial specifications). Blood samples were collected directly before the trial and immediately (within ∼5 min), 6 h, and 24 h after finishing the running trial. The day after the trial, all participants commenced 14 subsequent days of either the RIPC or SHAM procedure. After the final RIPC or SHAM procedure training day, all participants performed the same 20 × 400 m running trial, with blood collected before the trial and immediately (within ∼5 min), 6 h, and 24 h after finishing the trial. All blood samples were collected, and the serum was separated using a standard laboratory method, aliquoted, and frozen at −80°C until further analysis to evaluate the body’s response to RIPC training on post-exercise-induced changes in KYN metabolism. All laboratory analyses were performed at Gdansk University of Physical Education (Gdansk, Poland).

### 2.2 Participants

Semi-professional male marathon runners (n = 27) participated in this study. Runners were included in the study if they had completed a minimum of five marathons. The participants were randomly assigned to two groups: experimental (RIPC; n = 12) and control (placebo; SHAM; n = 15).

The basic characteristics of the study population are summarized in Table 1.

**TABLE 1:** Descriptive physical parameters before and after 14 days of RIPC/SHAM intervention.

Variable	Unit	RIPC group (n = 12)		SHAM group (n = 15)	
		Before	After	Before	After
		Mean ± SD	Mean ± SD	Mean ± SD	Mean ± SD
Body height	cm	180.00 ± 6.67	-	179.33 ± 7.09	-
Body mass	kg	78.9 ± 5.11	79.41 ± 4.12	79.05 ± 7.84	78.65 ± 9.14
Percent body fat	%	13.12 ± 4.44	13.35 ± 3.35	12.90 ± 6.00	13.21 ± 5.17
Body Mass Index	kg/m^2^	24.28 ± 4.65	24.62 ± 4.72	23.67 ± 2.96	23.32 ± 3.56

In accordance with the medical diagnostics and declaration, none of the runners had any history of known diseases or reported taking any medication due to illness in the 6 months leading up to the experiment.

All runners had at least five marathon history, within a finish time range of 175–210 min. One month before the start of the study, all participants refrained from alcohol and any other substances that could influence their exercise performance and affect the results (excluding caffeine, guarana, theine, and chocolate).

Two weeks before the study, after dietary consultation, all participants adopted a similar eating pattern. The dietary pattern for each individual was determined based on their sex, age, work, and physical activity.

The Bioethics Committee for Clinical Research of the Regional Medical Chamber of Gdansk approved the study protocol (consent no. KB 29/22), which was conducted in accordance with the Declaration of Helsinki. The trial was registered as a clinical trial (NCT05229835, date of first registration: 14/01/2022, direct link: https://classic.clinicaltrials.gov/ct2/show/NCT05229835).

All participants signed a written informed consent form to participate in the study and were informed about the study procedures; however, the participants were not informed about the study rationale and objectives to ensure that they were naive to the potential remote ischemic preconditioning effects.

### 2.3 Running trial of 20 × 400 m intervals

To induce the intracellular damage caused by physical exercise, interval training of 20 × 400 m with a 200 m active break for each run (jogging break time 75–80 s) was conducted at the beginning and end of the experiment. This is a classic training method used in long-distance running, which, owing to the nature and duration of the exercise, induces visible and significant physiological and biochemical changes. The running trial began at 10:00 a.m. at the Zawisza Athletic Stadium, Bydgoszcz, Kuyavian-Pomeranian Voivodeship, Poland.

During the trial, the temperature varied from 19°C to 21°C, the sky was clear (no visible clouds), and there was no wind. All experimental procedures were performed in September 2021. Weather conditions during both sessions were similar and did not affect the results of the running trials. Due to the varying athletic abilities of the participants in the study, a particular training module was used. The calculation was based on the best result for the 5 km run (performed 21 days before the main experiment).

Before starting the running trial, each participant received a timetable with individual times for each section. The time conditions of each subsequent running section were proportionally limited compared to the preceding one (Table 2).

**TABLE 2:** The scheme of interval training 20 × 400 m/200 m adapted to individual physical capabilities, along with sample assumptions for competitors with different times in the 5,000 m run.

5,000 m run time	Running pace	Average time of the 400 m sections	Running pace in sections 1–5	Running pace in sections 6–10	Running pace in sections 11–15	Running pace in sections 16–20
[min:s]	[min:s]	[s]	[s]	[s]	[s]	[s]
15	3:00	72	72–71	70–69	68–67	66–62
15:25	3:05	74	74–73	72–71	70–69	68–64
15:50	3:10	76	76–75	74–73	72–71	70–66
16:15	3:15	78	78–77	76–75	74–73	72–68
16:40	3:20	80	80–79	78–77	76–75	74–70
17:05	3:25	82	82–81	80–79	78–77	76–72
17:30	3:30	84	84–83	82–81	80–79	78–74
17:55	3:35	86	86–85	84–83	82–81	80–76
18:20	3:40	88	88–87	86–85	84–83	82–78
18:45	3:45	90	90–89	88–87	86–85	84–80
19:10	3:50	92	92–91	90–89	88–87	86–82
19:35	3:55	94	94–93	92–91	90–89	88–84
20:00	4:00	96	96–95	94–93	92–91	90–86
20:25	4:05	98	98–97	96–95	94–93	92–88
20:50	4:10	100	100–99	98–97	96–95	94–90
21:15	4:15	102	102–101	100–99	98–97	96–92
21:40	4:20	104	104–103	102–101	100–99	98–94
22:05	4:25	106	106–105	104–103	102–101	100–96
22:30	4:30	108	108–107	106–105	104–103	102–98
22:55	4:35	110	110–109	108–107	106–105	104–100
23:20	4:40	112	112–111	110–109	108–107	106–104
23:45	4:45	114	114–113	112–111	110–109	108–104
24:10	4:50	116	116–115	114–113	112–111	110–106
24:35	4:55	118	118–117	116–115	114–113	112–108
25:00	5:00	120	120–119	118–117	116–115	114–110

### 2.4 RIPC procedures

Each participant underwent 14 consecutive days of either RIPC or SHAM procedure to show its effects on post-exercise-induced changes in the kynurenine metabolism*.* In both cases, the procedure was performed at the same time (early morning) each day and under the control of colour flow Doppler ultrasound in the supine position, with bilateral arterial occlusion of both legs, according to Mieszkowski et al. (2021b). All participants, before and after any procedures, remained in a lying position for 10 min to normalize cardiac parameters.

The occlusion cuff was positioned proximally around the thigh and inflated to 220 mmHg (to block the arterial inflow) or 20 mmHg (placebo effect) in the RIPC and SHAM groups, respectively. Both procedures consisted of four sets of 5-min inflation, followed by 5-min deflation. The participants did not know the group allocation and differences in the procedures. Each time the procedure is performed, one participant and the researcher stay in the training room.

### 2.5 Sample collection and measurements of kynurenine metabolite levels

Blood samples were collected at four timepoints (directly before the trial and immediately, 6 h, and 24 h after the 20 × 400 m running trial) and placed into 5 mL BD Vacutainer Clot Activator Tubes (Becton Dickinson and Company, NJ, United States).

The serum was separated by centrifugation at 4,000 × *g* for 10 min and aliquoted into 500 μL portions. The samples were frozen and stored (≤3 months) at −80°C until further analysis.

KYN metabolites were measured using liquid chromatography coupled with tandem mass spectrometry, with minor modifications, as described by Midttun et al. (2009). Serum samples were analyzed in the positive ion mode using electrospray ionization. Raw data were collected, processed, and quantified using LabSolutions LCGC (Shimadzu Corporation, Kyoto, Japan). Various reagents were used for sample preparation. The derivatization reagent, 4-(4′-Dimethylaminophenyl)-1,2,4-triazoline-3,5-dione (DAPTAD), was synthesized by the Masdiag Laboratory (Warsaw, Poland). Water, ethyl acetate (Avantor Performance Materials Poland, Gliwice, Poland), and methanol (Honeywell; Sigma-Aldrich, Gillingham, Dorset, UK) were used as the solvents. Mobile phases were prepared using acetonitrile (Sigma-Aldrich; Merck KGaA Group, Darmstadt, Germany), water (Avantor Performance Materials Poland, Gliwice, Poland), and formic acid (Merck KGaA, Darmstadt, Germany). All the solvents used were of liquid chromatography-mass spectrometry grade.

The levels of the following KYN metabolites and amino acids were determined: phenylalanine (PHE), tryptophan (TRP), tyrosine (TYR), 3-Hydroxy-L-kynurenine (3-HK), anthranilic acid (AA), kynurenine (KYN), kynurenic acid (KYNA), quinolinic acid (QA) and xanthurenic acid (XANA). A Lactate Scout lactic acid analyzer (SensLab GmbH, Leipzig, Germany) was used to assess changes in lactate concentration during the trials. Capillary blood from the earlobe was collected by qualified medical personnel immediately before the start of the running trial and immediately after the run (to 3 min after the end of the last running trial). Blood samples for the determination (directly before and after the trial) of basic hematological parameters, creatine kinase (CK) and C-reactive protein (CRP) were analyzed by the accredited Synevo Laboratory (Bydgoszcz, Poland; PN-EN ISO 15189) using a hematological analyzer and an enzyme immunoassay method. Changes in systemic immune-inflammation index (SII index) were calculated as [Platelet [PLT] x Absolute Neutrophil [NEU]/Absolute Lymphocyte Counts [LY])/1,000] using the hematological parameters before and after 14-day of RIPC/SHAM intervention (Trifan and Testai, 2020).

### 2.6 Statistical analysis

The mean ± standard deviation (SD) for all measured variables was documented. The normality of distribution and the homogeneity of variance was checked by using the Shapiro–Wilk’s and Levene’s tests, respectively. As the assumptions of normality and homogeneity of variance were met, the analysis of variance (ANOVA) tests were used. A two-way ANOVA with repeated measures (2 × 4) was performed to investigate the impact of the 14-day remote ischemic preconditioning procedure on post-exercise-induced (20 × 400 m running trial) KYN metabolites (before the trial and immediately, 6 h, and 24 h after the trial) [group: RIPC, SHAM]. Another set of ANOVA with repeated measures (2 × 2) was performed to investigate the studied effect on performance, serum lactic acid, basic hematological parameters, CK, CRP and baseline values of the KYN metabolites (directly before and after the trial in RIPC and SHAM group).

In case of a significant interaction, Tukey’s *post hoc* test was performed to assess the differences in specific subgroups. In addition, the effect size was determined by eta-squared statistics (ƞ^2^). The values ≥0.01, ≥0.06, and ≥0.14 indicated small, moderate, and large effects, respectively. All calculations and graphics were performed using the Statistica 12 software (StatSoft, Tulsa, OK, United States). Differences were considered statistically significant at *p* < 0.05.

## 3 Results

When examining serum lactate and the physiological parameters by two-way ANOVA, no significant changes or group differences were observed before and after the 14-day RIPC/SHAM intervention (Table 3).

**TABLE 3:** Serum lactate and physiological parameters before and after 14 days of RIPC/SHAM intervention.

Variable	Unit	RIPC group (n = 12)		SHAM group (n = 15)	
		Before	After	Before	After
		Mean ± SD	Mean ± SD	Mean ± SD	Mean ± SD
AVG running time for 400 m	s	86.46 ± 6.8	82.62 ± 9.37	91.48 ± 11.05	82.12 ± 15.14
Maximal heart rate during 20 × 400 m running test	bpm	187.00 ± 6.69	185.21 ± 8.72	186.55 ± 6.42	181.22 ± 7.82
lactic acid serum concentration immediately after 20 × 400 m running test	mmol/L	11.21 ± 2.47	10.80 ± 1.91	11.60 ± 4.60	12.61 ± 3.49

The baseline values of the hematological and muscle damage parameters before and after the 14-day RIPC/SHAM intervention are presented in Table 4. Two-way ANOVA revealed a significant decrease in the baseline CK levels of the RIPC group after the 14-day RIPC intervention (35.2%, *p* < 0.01). In contrast, a slight increase in the baseline CK levels was observed in the SHAM group.

**TABLE 4:** Baseline values of hematological and muscle damage parameters before and after 14 days of RIPC/SHAM intervention.

Variable	Unit	RIPC group (n = 12)		SHAM group (n = 15)	
		Before	After	Before	After
		Mean ± SD	Mean ± SD	Mean ± SD	Mean ± SD
WBC	× 10^3^ μL	5.71 ± 1.02	6.11 ± 1.54	6.00 ± 1.83	5.65 ± 1.48
LY	× 10^3^ μL	2.12 ± 0.68	2.03 ± 0.54	2.15 ± 0.53	2.03 ± 0.54
MO	× 10^3^ μL	0.57 ± 0.13	0.59 ± 0.20	0.50 ± 0.11	0.45 ± 0.11
NEU	× 10^3^ μL	2.76 ± 0.59	3.12 ± 1.48	2.96 ± 1.40	2.75 ± 0.64
RBC	× 10^6^ μL	4.93 ± 0.30	4.94 ± 0.29	4.93 ± 0.27	4.96 ± 0.22
Hb	g/dL	14.15 ± 0.88	15.00 ± 0.77	15.34 ± 0.95	15.25 ± 0.61
Ht	%	42.86 ± 2.24	43.13 ± 2.57	42.57 ± 1.67	42.97 ± 1.76
MCV	fL	86.99 ± 2.70	87.40 ± 2.67	86.34 ± 2.92	86.53 ± 2.34
MCH	pg	30.52 ± 0.95	30.62 ± 1.03	30.63 ± 1.01	30.76 ± 1.04
MCHC	g/dL	35.17 ± 0.77	35.12 ± 0.63	35.45 ± 0.67	35.38 ± 0.68
Plt	× 10^3^ μL	233.57 ± 35.14	243.85 ± 34.16	250.09 ± 53.74	254.09 ± 55.85
MPV	fL	10.50 ± 1.01	10.17 ± 0.68	10.52 ± 1.03	10.67 ± 0.99
RDW	%	13.05 ± 0.50	13.65 ± 0.47	12.85 ± 0.49	12.82 ± 0.51
SII Index	×10^9^ cells/L	313.60 ± 98.13	334.03 ± 144.16	342.21 ± 165.67	346.61 ± 102.87
CK	U/L	281.69 ± 141.07	182.30 ± 90.33*	176.18 ± 68.43	195.63 ± 58.39
CRP	mg/L	0.88 ± 0.37	0.78 ± 0.45	1.06 ± 0.69	0.98 ± 0.62

Note: RIPC, remote ischemic preconditioning group; SHAM, sham-controlled group; WBC, white blood cells; LY, lymphocytes; MO, monocytes; NEU, neutrocytes; RBC, red blood cells; Hb, hemoglobin; Ht, hematocrit; MCV, mean corpuscular volume; MCH, mean corpuscular hemoglobin; MCHC, the mean concentration of corpuscular hemoglobin concentration; Plt, platelets; MPV, mean platelet volume; RDW, red blood cell distribution width; SII, Systemic immune-inflammation index; CK, creatine kinase; CRP, c-reactive protein.*Significant difference vs. before 14-days of RIPC/SHAM, intervention in RIPC, group at *p* < 0.01.

A change in the baseline serum concentration after 14 days of RIPC/SHAM intervention was observed only for phenylalanine (PHE) and the KYNA/KYN ratios. In the case of PHE, two-way ANOVA showed a significant increase in baseline concentration after 14 days of RIPC intervention (7.4%, *p* < 0.05), whereas in the SHAM group, there was a slight decrease. The analysis of the initial KYNA/KYN concentration showed a significant increase in the SHAM group (13.1%, *p* < 0.05), whereas no changes were noted in the RIPC group.

**FIGURE 1:**
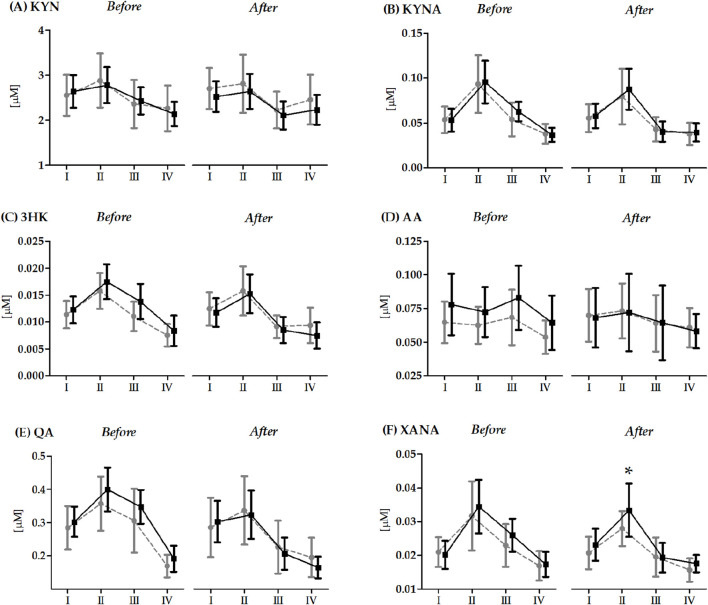
Changes in the serum kynurenine metabolite levels induced by a 20 × 400 m running test (RT) before and after the 14-day remote ischemic preconditioning/control intervention (mean and standard deviation). Abbreviations/Symbols: KYN **(A)**, kynurenine; KYNA **(B)**, kynurenic acid; 3-HK **(C)**, 3-hydroxy-L-KYN; AA **(D)**, anthranilic acid; QA **(E)**, quinolinic acid; XANA **(F)**, xanthurenic acid; RIPC, remote ischemic preconditioning group; SHAM, sham control group; I, before the 20 × 400 m RT; II, immediately after the 20 × 400 m RT; III, 6 h after the 20 × 400 m RT; IV, 24 h after the 20 × 400 m RT. Gray color indicates the RIPC group (n = 12); black color indicates the SHAM group (n = 15). * Significant difference vs. RIPC group immediately after the 20 × 400 m RT. The significance level was set at *p* < 0.05.

Changes in KYN metabolite levels induced by the 20 × 400 m running test before and after the 14-day RIPC/SHAM intervention are presented in Figure 1. The 20 × 400 m RT significantly impacted all KYN metabolites tested, both before and after the 14-day RIPC/SHAM intervention. Regardless of the group factor, immediately after the 20 × 400 m RT, a significant increase in the serum concentration levels of 3-HK (before RIPC/SHAM intervention 29.9%, *p* < 0.01; after RIPC/SHAM intervention 21.9%, *p* < 0.01), KYN (before RIPC/SHAM intervention 9.2%, *p* < 0.01), KYNA(before RIPC/SHAM intervention 43.6%, *p* < 0.01; after RIPC/SHAM intervention 31.7%, *p* < 0.01), QA (before RIPC/SHAM intervention 21.6%, *p* < 0.01; after RIPC/SHAM intervention 11,0%, *p* < 0.01), and XANA (before RIPC/SHAM intervention 45.6%, *p* < 0.01; after RIPC/SHAM intervention 35.5%, *p* < 0.01) were observed in comparison to the baseline values. Moreover, 24 h after the run, the concentration levels of these metabolites and AA were significantly lower than the baseline values. A significant group effect (F = 7.22, *p* < 0.01, η^2^ = 0.22) and interactions between the group factor and RT (F = 3.01, *p* < 0.05, η^2^ = 0.11) were detected solely in XANA after the 14-day RIPC/SHAM intervention. Post-hoc analysis showed a significantly lower concentration of XANA in the RIPC group compared to the SHAM group immediately after the RT (26.4%, *p* < 0.05).

**FIGURE 2:**
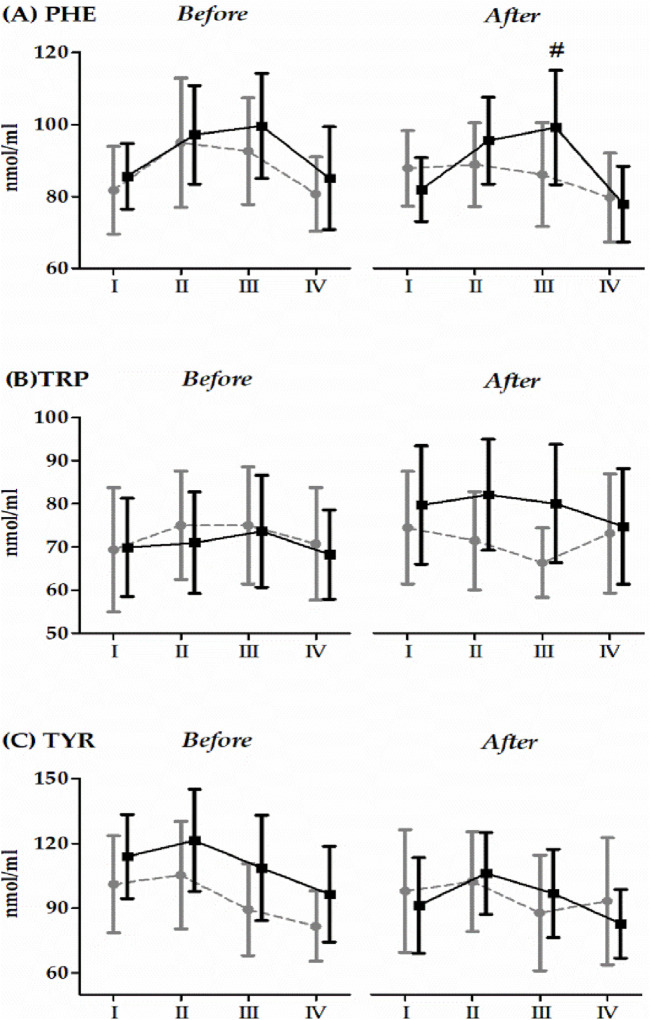
Changes in the serum levels of phenylalanine, tryptophan and tyrosine induced by a 20 × 400 m running test (RT) before and after the 14-day ischemic preconditioning intervention (mean and standard deviation). Abbreviations/Symbols: PHE **(A)**, phenylalanine; TRP **(B)**, tryptophan; TYR **(C)**, tyrosine; RIPC, remote ischemic preconditioning group; SHAM, sham control group; I, before the 20 × 400 m RT; II, immediately after the 20 × 400 m RT; III, 6 h after the 20 × 400 m RT; IV, 24 h after the 20 × 400 m RT. Gray color indicates the RIPC group (n = 12); black color indicates the SHAM group (n = 15). # Significant difference vs. before and 24 h after the 20 × 400 m RT in SHAM group. The significance level was set at *p* < 0.01.

Changes in the serum levels of phenylalanine (PHE), tryptophan (TRP), and tyrosine (TYR) induced 20 × 400 m running test (RT) before and after 14 days RIPC/SHAM intervention was shown in Figure 2. Two-way ANOVA demonstrated that the RT significantly affected PHE and TYR both before and after the 14-day RIPC/SHAM intervention. In the case of PHE, a significant increase was noted immediately after (before RIPC/SHAM intervention, 13.1%, *p* < 0.01) and 6 h after the RT (before RIPC/SHAM intervention, 12.8%, *p* < 0.01; after RIPC/SHAM intervention, 7.3%, *p* < 0.01), when compared with the baseline values. ANOVA also revealed in PHE a significant interaction between the group factor and repeated measures (F = 3.29, *p* < 0.05, η2 = 0.12) after the 14-day RIPC/SHAM intervention. Post-hoc results demonstrated a significant increase in PHE concentration in the SHAM group 6 h after the RT compared to the baseline values (17.1%, *p* < 0.05); contrastingly, no significant changes were observed in the RIPC group. TRP concentration increased significantly 6 h after the RT, before the 14-day RIPC/SHAM intervention (44.2%, *p* < 0.01). Conversely, 14 days after the RIPC/SHAM intervention, the group factor (F = 5.67, *p* < 0.05, η^2^ = 0.27) significantly impacted the TRP concentrations, whereby a significantly lower TRP concentration was observed in the RIPC group compared to the SHAM group (10.0%, *p* < 0.01). The lack of a significant impact from the RT may have resulted in the downward trend observed in the TRP concentration of the RIPC group (Figure 2). In the case of TYR, after a slight increase in its concentration immediately after the run, there was a significant decrease 6 h after (before RIPC/SHAM intervention 8.5%, *p* < 0.05) and 24 h after the 20 × 400 m RT (before RIPC/SHAM intervention 21.7%, *p* < 0.01; after RIPC/SHAM intervention 14.9%, *p* < 0.05).

**FIGURE 3:**
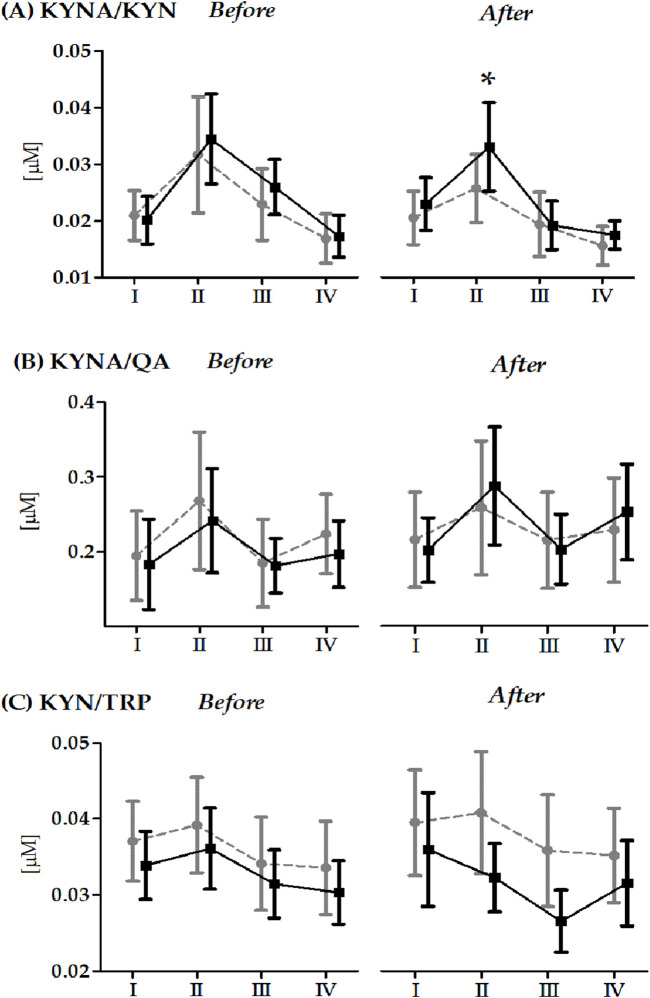
Changes in the kynurenine metabolite and tryptophan ratios induced by a 20 × 400 m running test (RT) before and after the 14-day ischemic preconditioning intervention (mean and standard deviation). Abbreviations/Symbols: KYNA/KYN **(A)** kynurenic acid/kynurenine ratio; KYNA/QA **(B)**, kynurenic acid/quinolinic acid ratio; KYN/TRP **(C)**, kynurenine/tryptophan ratio; RIPC, remote ischemic preconditioning group; SHAM, sham control group; I, before the 20 × 400 m RT; II, immediately after the 20 × 400 m RT; III, 6 h after the 20 × 400 m RT; IV, 24 h after the 20 × 400 m RT. Gray color indicates the RIPC group (n = 12); black color indicates the SHAM group (n = 15). * Significant difference vs. RIPC group immediately after the 20 × 400 m RT. The significance level was set at *p* < 0.05.

Changes in kynurenine metabolite and tryptophan ratios induced 20 × 400 m RT before and after 14 days of RIPC/SHAM intervention presented in Figure 3. Regardless of the group, there was a significant increase in the KYNA/KYN ratio (before RIPC/SHAM intervention, 33.3%, *p* < 0.01; after RIPC/SHAM intervention, 30.0%, *p* < 0.01), KYNA/QA (before RIPC/SHAM intervention, 25.9%, *p* < 0, 01; after RIPC/SHAM intervention, 22.4%, *p* < 0.01) immediately after the 20 × 400 m RT compared to the baseline values. After the experiment, there was a significant decrease in KYN/TRP concentration at 6 h (13.5%, *p* < 0.01) and 24 h (14.4%, *p* < 0.01) after the 20 × 400 m RT compared to the low increase noted immediately after the run. In contrast, after the experiment, the significant effect of the group factor (F = 5.96, *p* < 0.05, η^2^ = 0.19) was significantly lower regarding KYN/TRP values in the SHAM group compared to the RIPC group (18.4%, *p* < 0.05). After the experiment, a significant interaction between the group and repeated measurement (F = 3.34, *p* < 0.05, η^2^ = 0.12) in KYNA/KYN was also noted. Post-hoc results demonstrate significantly lower values in the RIPC group immediately after the 20 × 400 m RT compared to the SHAM group (15.1%, *p* < 0.05).

## 4 Discussion

We previously showed that ultramarathons induce significant changes in serum KYN levels and that vitamin D modifies TRP metabolism during exercise (Mieszkowski et al., 2022). In the present study, we confirmed that strenuous interval exercise (20 × 400 m) also induces significant changes in metabolism. We observed that KYN, KYNA, QA, PA, and 3-HK increased after exercise, and RIPC did not affect these changes. Remote ischemic preconditioning of skeletal muscles can exert beneficial effects on other tissues. In our previous study, we demonstrated that remote ischemic preconditioning changes serum exerkine levels and reduces inflammation. This may be an effective method to reduce marathon-induced oxidative stress and decrease liver and heart injury markers (Mieszkowski et al., 2021a).

Some of the TRP metabolites may present neuroprotective or neurotoxic activity (neuro-protective [kynurenic acid (KYNA), picolinic acid (PA) and xanthurenic acid (XANA)] and neurotoxic [quinolinic acid (QA), 3-hydroxykynurenin (3-HK)] (Agudelo et al., 2018; Jung et al., 2020). The synthesis of specific kynurenine pathway metabolites is tightly regulated and may considerably vary under physiological and pathological conditions. Therefore, understanding the effects of RIPC on TRP metabolism may allow for a better understanding of the mechanisms underlying its effects. Here, we observed that XANA was the only metabolite whose blood concentration after exercise was affected by RIPC. It is important to note that XANA can penetrate the blood-brain barrier (Fukui et al., 1991). XANA has been shown to have beneficial impacts on the central nervous system such as stimulating dopamine release in the cortex and striatum (Taleb et al., 2021). We speculate that the lower concentration of XANA observed in athletes who underwent 14 days of RIPC training may be attributed to an increase in its absorption by the brain. XANA is formed from 3-HK during a reaction that is catalyzed by KATs. The same enzymes are responsible for KYNynA formation. Exercise-induced changes in the serum concentrations of 3-HK and KYNA were the same in both the SHAM group and RIPC-trained athletes. Thus, it can be assumed that RIPC training does not influence XANA formation but rather its transport into the target tissue (brain). Conversely the ratio of KYNA/KYN indicates activity of KAT an enzyme that can be upregulated by endurance exercise training (Agudelo et al., 2014). Here we observed that the ratio was lower after exercise in RIPC-trained athletes. The meaning of this changes are elusive as we have not measured the activity of KATs in skeletal muscle after RIPC training.

Changes in the KYNA/QA and KYN/TRP ratios induced by exercise were the same in both SHAM group and RIPC-trained athletes. Former is regarded “neuro-protective” because of the anticipated neuroprotective properties of KYNA and the neurotoxic properties of QA and the later reflects activity of IDO (Cervenka et al., 2017).

Another important aspect evaluated in this study was the effect of RIPC training on exercise-induced changes in serum TRP levels. As most KYNs increase after exercise, TRP levels are expected to decrease. TRP and KYN have been shown to decrease in the circulatory system following acute endurance exercise (Schlittler et al., 2016; Isung et al., 2021). Here, we observed that after exercise, serum TRP did not change; however, after RIPC training, its concentration remained below that of the control group. The effect of RIPC training on TRP metabolism, therefore, remains unclear. After the 14 days of RIPC training, there was visible, yet nonsignificant decrease in serum TRP levels after the run in the RIPC group. During exercise, serum TRP is taken up by various cells and metabolized to KYN and other metabolites. Several serum TRP metabolites increase after exercise, explaining some decreases observed in TRP levels. However, the decrease in TRP observed in the RIPC group cannot be explained by differences in KYN formation. During long-term exercise, more skeletal muscle proteins undergo proteolytic degradation, and more amino acids are exported into the blood. Endurance exercise increases the expression of Atrogin-1, which is known to stimulate skeletal muscle protein degradation (Stefanetti et al., 2015).

Conversely, RIPC inhibits sarcolemma protein proteolysis via matrix metalloprotease-2 (MMP-2) inhibition (Bin et al., 2023). Hence, skeletal muscle proteolysis may be partially inhibited in athletes after RIPC training. Our data supports this assumption. First of all, CK, a marker of skeletal muscle damage, was significantly lower after RIPC training, indicating protection, whereas no changes were observed in the SHAM group. In addition, changes in the serum levels of the aromatic amino acid PHE in the SHAM group were distinct from those of the RIPC-trained group. PHE is not degraded by skeletal muscle and can, therefore, be used to indicate the balance between protein synthesis and degradation. We observed a significant increase in serum PHE levels in the SHAM group and no change in the RIPC group, indicating higher proteolysis in the control group. The observed decrease in the serum TRP levels after exercise in the RIPC group may have positive implications. Serotonin is formed from TRP in the central nervous system, and this process is regulated by serum TRP availability (Patrick and Ames, 2014). A decrease in serum TRP levels can lead to reduced serotonin formation, which can reduce exercise-induced fatigue (Yamamoto and Newsholme, 2000; Yamamoto and Newsholme, 2003). A notably higher KYN/TRP ratio in the RIPC group compared to SHAM suggests increased activation of IDO activity (Badawy and Guillemin, 2019). We assume that this observation is not coincidental but is a rather consequence of differences in TRP concentration after the run.

In conclusion, our data demonstrate that repeated RIPC intervention in the form of training modifies exercise-induced changes in XANA, PHE, and TRP. These changes could have significant physiological effects and should be studied further in the context of brain health, cognitive function and central fatigue.

## 5 Limitations of the study

The current study has some potential limitations. The first limitation is the relatively small sample size. Further studies should be performed, with an increased sample size and/or implementing a crossover study design. Second, the study did not include a specific control of the dietary regime, and potential changes in the amount of consumed dietary products might have interfered with the applied intervention. However, it should be mentioned that 2 weeks before the study, after dietary consultation, all participants were asked to adopt a similar eating pattern determined by their sex, age, work, and physical activity. So, we conclude that the effect of this factor on achieved results should be limited but not entirely forgotten and discarded.

The third limitation, as highlighted by previous studies applying this specific remote ischemic procedure, was the challenge of conducting a truly double-blind study. The issue arises because the SHAM intervention does not induce perceptible pain, making it difficult to maintain indistinguishable conditions between the experimental groups. Mieszkowski et al. (2021b) proposed that a 20-mmHg compression in the SHAM group allows participants to experience external tissue pressure, yet this does not lead to sensations of pain or even discomfort during procedure performance.

### 5.1 Practical applications and further research

This study provides insight into the metabolic effects of using RIPC as a sport enhancement method. Although it was established in previous studies that RIPC could improve inflammation status and modify post-exercise tissue disfunction, none of them assessed the direct effect of this training method on kynurenine metabolism. Our findings provide valuable information that RIPC modulates post-exercise changes in XANA and TRP levels, which can be beneficial for brain health, yet further researches are needed to establish the direct mechanism of observed action and how this type of stimulus affects tissues over a more extended period.

Further clinical evaluation is required to determine whether RIPC can improve clinical outcomes and brain health not only in healthy populations but also in the case of patients suffering from many ischemic tissue injuries and problems (post-stroke etc.). Such observations and results could contribute to a change in clinical practice and widely use of this training procedure in different age and health populations.

## Data Availability

All original contributions presented in the study are included in the article/supplementary material, further inquiries can be directed to the corresponding authors.
